# Internet-Of-Things in Motion: A UAV Coalition Model for Remote Sensing in Smart Cities

**DOI:** 10.3390/s18072184

**Published:** 2018-07-06

**Authors:** Adiel Ismail, Bigomokero Antoine Bagula, Emmanuel Tuyishimire

**Affiliations:** Department of Computer Science, University of the Western Cape, Private Bag X17, Bellville 7535, South Africa; abagula@uwc.ac.za (B.A.B.); tuyinuel@gmail.com (E.T.)

**Keywords:** smart cities, Internet-of-Things, multi-drone task allocation, unmanned aerial vehicles, path planning, Dubins curves, particle swarm optimization

## Abstract

Unmanned aerial vehicles (UAVs) or drones are increasingly used in cities to provide service tasks that are too dangerous, expensive or difficult for human beings. Drones are also used in cases where a task can be performed more economically and or more efficiently than if done by humans. These include remote sensing tasks where drones can be required to form coalitions by pooling their resources to meet the service requirements at different locations of interest in a city. During such coalition formation, finding the shortest path from a source to a location of interest is key to efficient service delivery. For fixed-wing UAVs, Dubins curves can be applied to find the shortest flight path. When a UAV flies to a location of interest, the angle or orientation of the UAV upon its arrival is often not important. In such a case, a simplified version of the Dubins curve consisting of two instead of three parts can be used. This paper proposes a novel model for UAV coalition and an algorithm derived from basic geometry that generates a path derived from the original Dubins curve for application in remote sensing missions of fixed-wing UAVs. The algorithm is tested by incorporating it into three cooperative coalition formation algorithms. The performance of the model is evaluated by varying the number of types of resources and the sensor ranges of the UAVs to reveal the relevance and practicality of the proposed model.

## 1. Introduction

Drone technology has recently moved from a niche area mainly controlled by the American Department of Defense into a technology that can be accessed off-the-shelf for different purposes and tasks. Presently, drones are increasingly used for tasks that are either too dangerous, expensive or difficult for human beings [1]. Drones are also used in cases where a task can be performed more economically or more efficiently than if done by humans. Target search is a common application widely used in missions involving rescue [2,3], monitoring [4,5] or destruction [6,7]. Area coverage is also a popular application for multiple purposes, which include environment mapping [8,9], surveillance [4,10,11,12,13,14], sensor deployment [15,16], aerobiological sampling [17] or acting as communications hubs for immobile wireless sensor networks [15,18]. UAVs can also be used to provide data muling services in applications such as smart parking [19,20] and drought mitigation [21]. Other applications include fire detection, weather forecasting, space exploration, traffic surveillance, environmental clean-up, agricultural monitoring and interior surveillance of buildings. New and unforeseen applications also continue to surface. In Israel and Australia, UAVs are being used to search for groundwater. More recently, commercial applications using drones have emerged in niche areas where these vehicles have been found much more practical and more economically sound than traditional applications. These include cases where drones are being used commercially: (1) as marketing gimmicks, (2) to deliver pizza by Domino’s pizza in the U.S., (3) to deliver flowers on Valentine’s Day and (4) to deliver beer during a festival. Drones equipped with cameras have been deployed to help combat the scourge of rhino-poaching on game farms in Africa, while the Australian defense force has started using UAVs to monitor its vast coastline. Facebook and Google have recently acquired aerospace companies Ascenta and Titan Aerospace, respectively, and are investing in drones for supplying remote areas on Earth with broadband and Internet connectivity. It is envisaged that their solar-powered drones will be capable of staying in the air for months and beam WiFi signals to inaccessible areas of the world. In 2014, Alec Momont, in the Netherlands, developed an ambulance drone that delivers an automatic external defibrillator to patients via air, much faster than a standard ambulance [22]. This invention could bring relief to many of the approximately 360,000 patients who experienced cardiac arrest in the United States last year. These applications along with the potential for drones to deliver life-saving medicines and supplies to isolated communities in rural and disaster zones when overland access is not an option make the multi-drone task allocation an interesting research area that may benefit both urban and rural areas of the world. The use of a team of drones instead of a single drone is possible when the mission to be performed can be decomposed into independent tasks, i.e., the mission ‘is inherently distributed in terms of space, time and functionality’ [23]. However, path finding and resources allocation, both aerial and ground-based, are two key challenging issues that need to be addressed in order to provide a team of drones with the necessary autonomy to achieve a task cooperatively.

### 1.1. Related Work

In [24], Dubins presented a solution for the shortest path between two positions given a car’s direction or heading at both positions. His solution consists of all combinations of arcs of minimal turning radius of the car and a straight line segment joining the two arcs. The shortest path solution provided by Reeds and Shepp in [25] included both forward and backward movement of the vehicle. In [26], Kavraki et al. constructed a probabilistic road map of all feasible paths between the source and target, and the path with the lowest cost was then selected as a solution. In [27], a swarm of UAVs with airborne sensors cooperated in path planning by using Dubins curves in conjunction with splinegons, which are generalizations of polygons with sides of constant curvature, to detect the boundaries of contaminant clouds in order to model and track their movement. In [28], a path generator was proposed that finds the shortest path for a fixed-wing UAV from its current position and direction of flight to a new position and direction. The generated path was traversed using their Lyapunov path-following scheme. In [29], a path planning approach for mobile robots was modeled as an optimization problem with the objective of achieving a smooth path linking several waypoints by using fifth order Bézier curves. In [30], a path planning model was proposed for many UAVs to cooperatively collect data at different locations. A particle swarm optimization (PSO) based distributed coalition auction algorithm for real-time task assignment that uses a dynamic bidding strategy to address target assignment conflicts between UAVs, which resulted in shorter mission completion times, while still maintaining a high mission completion rate, was proposed in [31] by Lin et al. In [32], Fargeas et al. used a heuristic algorithm to plan the paths of a team of UAVs patrolling a network of roads and pursuing intruders detected by unattended ground sensors where UAV paths were scheduled using revisit deadlines that resulted in most of the intruders being identified. In [33], Kwok et al. used PSO to determine drive commands, speed and turning, to coordinate the motion of construction vehicles for task assignment by minimizing the distance and difference in orientation between the current positions of the vehicles and the positions of the targets. Collisions between vehicles were avoided using a behavioral-based reactive approach in conjunction with a dynamical indexing schedule that reduced dead-lock events. Bellingham et al. in [34] solved large problems involving many obstacles and waypoints by partially decoupling the task allocation and trajectory design aspects of the fleet coordination problem. Completion times for the various allocation scenarios were efficiently estimated and then passed on to the allocation problem that was solved using mixed-integer linear programming. In [35], Tong et al. successfully applied a cooperative control strategy based on Voronoi diagrams and discrete PSO to cooperative-trajectory planning problems with timing constraints. Path planning and task allocation are crucial aspects in the application of a fleet of UAVs to remote sensing. UAVs collecting data directly from targets close to each other often result in non-optimal flight paths due to the limitation of a UAV’s minimum turning radius. In [36], a UAV path planner was presented that collected sensing data using a minimal distance by exploiting the sensor footprint and by pruning a tree of feasible paths, which was based on the learning real-time A* search algorithm. Road network search was defined as a graph in [37] and first solved as a Chinese postman problem and then modified to address the physical constraints of UAVs before using a multiple multidimensional knapsack in conjunction with Dubins paths to find the shortest flight path. In [38], task allocation was decentralized, and three complementary algorithms based on swarm intelligence and multi-agent systems were presented, which outperformed current approaches. In [39], a UAV’s flight path was adapted based on fluctuations detected for several performance parameters, and the path planner calculated an optimal flight path by considering: avoidance of obstacles using a probabilistic risk model, the limitation of the maneuverability of a UAV and the velocity and flight time to a target.

### 1.2. Contributions and Outline

**Figure 1:**
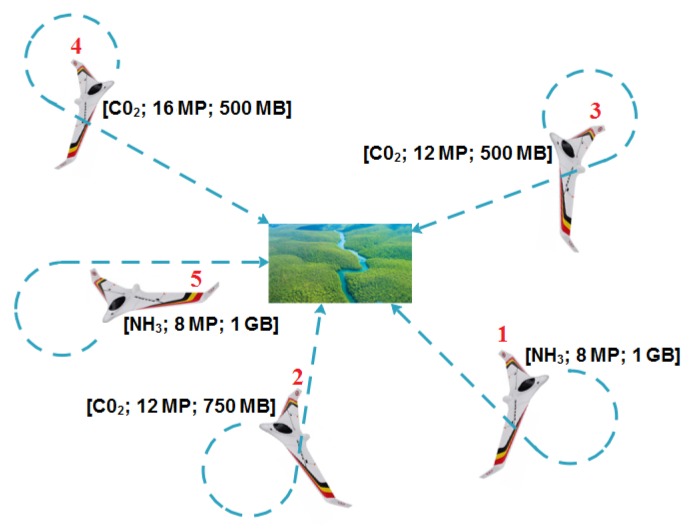
Visual sensing and pollution monitoring scenario.

This paper proposes a collaborative strategy and mechanisms to control multiple UAVs and sensor networks with the objective of surveillance and achieving remote sensing in a city. A potential deployment scenario for the model presented in this paper consists of remote sensing in a city where different environment parameters need to be collected by a team of drones at separate locations of interest. The environment sensing parameters may differ from location to location, and different drones can be equipped with different sensing resources, thus leading to a model where drones need to form coalitions in order to meet the sensing requirements of different locations. The remote sensing services may consist, for example, of (i) pollution monitoring of different pollutants to be measured at different locations of the city and (ii) visual sensing by a number of drones coming from different angles to capture a view of a scene of a given location as depicted in Figure 1. In this scenario example, the pollution monitoring parameters and visual information captured at different locations will be ferried to a processing place where the different portions of the scene are stitched together to reproduce the whole scene of the location, and levels of pollution are evaluated at the location using machine learning techniques.

The main contributions of this paper include:

1. An algorithm to calculate the Dubins longest path using elementary geometry is presented.
2. For successful collection of environment parameters from various locations, agents need to have sufficient storage capacity that can accommodate all the parameters. Agents in the experiments in this paper are equipped with sufficient storage capacity that can accommodate all the environment parameters collected from the various locations.
3. The effect of the increase of the number of types of environment parameters on the performance of the algorithms is investigated.
4. The effect of the increase of the sensor range on the performance of the algorithms and mission completion time are also investigated.
5. The energy gain of the proposed approach in (2) is quantified.

This paper borrows from Manathara et al. in [40] the three coalition formation algorithms, but uses a different model where the mission completion time is used as cost for minimization, while Manathara et al. used total coalition path length as the cost. In addition, the model introduced in this paper uses agents that were initialized with sufficient resources that met all the targets’ resource requirements, would lead to complete prosecution of all the targets. The path generation was introduced in [41].

The remainder of this paper is organized as follows. The coalesced city’s remote sensing model is described in Section 2. Section 3 describes the problem statement and algorithms used in this paper. The approach to calculate the Dubins longest path is described in Section 4. Experiments and results are reported in Section 5. The paper is concluded in Section 6.

## 2. The Coalesced City’s Remote Sensing Model

### 2.1. Model Formulation

Let us consider a hybrid sensor network represented by a directed graph G=(N,L,S,Q), where N is the set of cluster sensor nodes, L is the set of locations of interest in a given region, S is the set of the UAV base stations, while Q is the set of UAVs. Consider the case where each location has to be visited only once by a set of UAVs at time r(i) and a set of UAVs forming a coalition $C_{oal}\left(i\right)$ spend an amount of time t(i) to arrive at the location *i* with a risk ϵ(i) of arriving earlier than planned or after the expected arrival time. Assume that $D\left(G\right)=\left\{D_{n}\right\}$ is the set of the city’s remote sensing configurations associated with the hybrid network G.

### 2.2. The City’s Remote Sensing Problem

The coalesced city’s remote sensing task is based on some rules, which can be expressed in terms of constraints and/or objectives to be met by the coalition of UAVs and resources needed at the specific locations. These rules are described as follows:

- **Visiting time at locations:** An early visit to a location will be penalized, and a late visit to a location is also penalized. Therefore, a visit by a UAV k(i)∈Q, which is part of a coalition $C_{oal}\left(i\right)$ at location *i*, will be constrained by:
  (1) $$0\leq r\left(i\right)-t\left(i,k\left(i\right)\right)<T_{1},\;\;\forall \;\;k\left(i\right)\in C_{oal}\left(i\right)i\;\mathrm{and}$$
  (2) $$T_{2}<r\left(i\right)-t\left(i,k\left(i\right)\right)<0,\;\;\forall \;\;k\left(i\right)\in C_{oal}\left(i\right),$$
  where t(i,k(i)) is the time taken by UAV k(i) to arrive at location *i*. $T_{1}$ and $T_{2}$ are the allowed early and late thresholds, respectively. The model considers that there is a penalty assigned to the UAV if it arrives early t(k(i))<r(i) and late t(k(i))>r(i).
- **Resource requirements at locations:** The sum of resources required by a UAV coalition at location *i* is constrained by:
  (3) $$\sum_{i\in C_{oal}\left(i\right)}R_{src}^{c}\left(k\left(i\right)\right)\geq R_{eq}^{c}\left(i\right),\;\;\forall \;\;c\in \left\{1,2,\ldots ,C\right\},$$
  where $R_{src}^{c}\left(k\left(i\right)\right)$ is the resource of type *c* carried by UAV *k* and *C* the total number of resources at the location of interest.
- **UAV teams’ coalition:** A data collection model is proposed where a coalition of UAVs $C_{oal}\left(i\right)$ collects data at a given location *i*. Each network configuration $D_{n}$ is defined by a partition of the set of UAVs Q and made up of a set of UAV coalitions at different locations as expressed by:
  $$\underset{i\in L}{⋃}C_{oal}\left(i\right)=Q\;\mathrm{and}$$
  $$|C_{oal}\left(i\right)|=\sum_{k\in Q}y\left(i,k\left(i\right)\right),$$
  where $|C_{oal}\left(i\right)|$ is the size of a coalition at node i and y(i,k(i)) defined by:
  (4) $$y\left(i,k\left(i\right)\right)=\left\{\begin{array}{cc} 1, & \mathrm{if}\;k\left(i\right)\in C_{oal}\left(i\right),\\ 0, & \mathrm{otherwise}. \end{array}\right.$$
  This coalition process is constrained by the fact that each UAV can be either part of only one coalition as expressed by:
  (5) $$C_{oal}\left(i\right)\cap C_{oal}\left(j\right)=\emptyset ,\;\forall i,j\in L,$$
  or of more than one coalition if it has enough resources as expressed by:
  (6) $$C_{oal}\left(i\right)\cap C_{oal}\left(j\right)\neq \emptyset ,\;\mathrm{if}\;R_{src}\left(k\left(i\right)\right)\geq R_{eq}\left(i\cup j\right).$$
- **The city’s remote sensing problem** consists of finding a cost-efficient city remote sensing configuration $D_{cef}$, such that:
  (7) $$\begin{array}{ccc} {\hat{\tau }}_{cst}\left(D_{cef}\right) & = & \min_{D_{n}\in D\left(G\right)}\tau_{cst}\left(D_{n}\right),\\ & = & \min_{D_{n}\in D\left(G\right)}\sum_{i\in L}y\left(i,k\left(i\right)\right)\left(\max_{C_{oal}\left(i\right)\in D_{n}}D_{ist}\left(k\left(i\right)\right)\right),\;\forall k\left(i\right)\in C_{oal}, \end{array}$$
  subject to the constraints:
  (8) $$\begin{array}{c} 0\leq r\left(i\right)-t\left(i,k\left(i\right)\right)<T_{1},\;\;\forall \;\;k\left(i\right)\in C_{oal}\left(i\right) \end{array}$$
  (9) $$\begin{array}{c} T_{2}<r\left(i\right)-t\left(i,k\left(i\right)\right)<0,\;\;\forall \;\;k\left(i\right)\in C_{oal}\left(i\right) \end{array}$$
  (10) $$\begin{array}{c} k\in C_{oal}\left(i\right)\cap C_{oal}\left(j\right)\;\mathrm{if}\;R_{src}\left(k\left(i\right)\right)\geq R_{eq}\left(i\cup j\right) \end{array}$$
  (11) $$\begin{array}{c} \underset{i\in L}{⋃}C_{oal}\left(i\right)=D_{cef} \end{array}$$
  (12) $$\begin{array}{c} \sum_{i\in C_{oal}\left(i\right)}R_{src}^{c}\left(k\left(i\right)\right)\geq R_{eq}^{c}\left(i\right),\;\;\forall \;\;c\in \left\{1,2,\ldots ,C\right\} \end{array}$$
  (13) $$\begin{array}{c} y\left(i,k\left(i\right)\right)=\left\{\begin{array}{cc} 1, & \mathrm{if}\;k\left(i\right)\in C_{oal}\left(i\right),\\ 0, & \mathrm{otherwise}, \end{array}\right. \end{array}$$
  (14) $$\begin{array}{c} R_{eq}\left(i\right)=\sum_{s\in S}R_{eq}\left(k\left(i\right),s\right), \end{array}$$
  where $D_{ist}\left(k\left(i\right),i\right)$ is the distance covered by UAV k(i) when forming part of coalition $C_{oal}\left(i\right)$ to reach location *i*. Note that the constraints (8) and (9) are timing constraints defining respectively the arrival requirements at location *i* and the city’s remote sensing scheduling at the same location. Equations (10)–(13) are resource allocation constraints. Note also that the constraints  (10), (11) express the fact that one UAV can participate in different coalitions if it has remaining resources after a first coalition. These constraints could be relaxed to restrict a UAV to participate in only one coalition as expressed by:
  (15) $$\begin{array}{c} k\in C_{oal}\left(i\right)\cap C_{oal}\left(j\right)\;\mathrm{if}\;R_{src}\left(k\left(i\right)\right)\geq R_{eq}\left(i\cup j\right) \end{array}$$
  (16) $$\begin{array}{c} \underset{i\in L}{⋃}C_{oal}\left(i\right)=D_{cef} \end{array}$$

### 2.3. The City’s Remote Sensing Process

**Figure 2:**
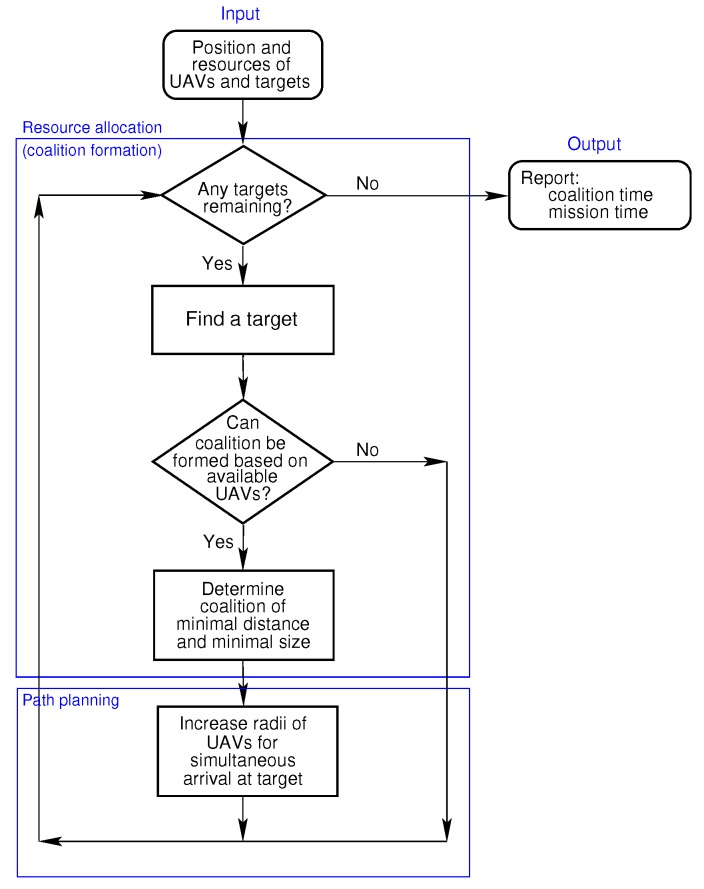
Diagram depicting solution steps.

At the start of the process, the positions of the locations of interest and the resources on-board the UAVs are provided. The solution presented in Figure 2 consists of two steps,

1. Resource allocation or coalition formation:
  Each UAV may contain three types of resources, i.e., a pollutant sensor, a camera and on-board storage. If all the targets or locations of interest have been visited, then execution details, such as the coalition and mission times, are reported, otherwise proceed and find any of the remaining locations of interest. If the combined resources of the available UAVs that are not assigned to coalitions fulfil the resource requirement of the location of interest, then proceed to form a coalition of minimal distance each time, by including from the available UAVs the one closest to the location of interest in the potential coalition until the resource requirement of the location of interest is met.
2. Path planning:
  Once a coalition is formed, the radii of the coalition members are increased to ensure that their Dubins distances are all equal to that of the UAV furthest from the location of interest under consideration. The coalition members proceed to fly to the location of interest. After collecting the sensor data at the location of interest, the UAVs become available to form further coalitions should they have any resources remaining.

## 3. Problem Statement and Algorithms

A number of fixed-wing UAVs are required to collect different types of environment parameters from various locations. Each location may contain different amounts of each of the different types of environment parameters. Each UAV is capable of storing each type of environment parameter, but in different quantities. For example, a UAV may contain a sensor to detect the levels of pollutants such as ${\mathrm{CO}}_{2}$ or ${\mathrm{NH}}_{3}$, a camera with picture quality ranging from 8–16 megapixels (MP) and on-board storage ranging from 500 megabytes (MB) to two gigabytes (GB). See the illustration in Figure 1. The UAVs do not know the positions of the locations of interest and fly in search of it and can only detect a location’s position once it is within the UAVs’ sensor range. All UAVs can communicate with each other. Once the position of a location of interest is detected, a single-member coalition or multiple-member coalition can potentially be formed to collect the environment data using Dubins longest path. After collecting the data at a location, the UAVs that formed part of the coalition are released and continue to search for positions of the remaining locations of interest and may participate in further coalitions if they have any data storage space available. The objective of coalition formation is to complete a mission in minimum time, by forming a coalition for each location of interest that (a) reaches the position of a location in minimum time, (b) uses a minimum number of UAVs and (c) collects the environment data simultaneously.

In [40], the combined resources of all UAVs may not be sufficient to prosecute all the targets. This will be the case if for each resource type, the sum of the quantities of the resource under consideration of all UAVs is not equal to, or does not exceed, the sum of the quantities of the same type of resource of all the targets. No test is performed initially to check whether the combined resources of all UAVs are sufficient to prosecute all the targets. Instead of initiating a mission that may not end in success, one can establish if the combined storage capacity of all UAVs in a mission is adequate to accommodate the environment data of all locations of interest before commencing a mission. For the data used in the experiments reported in this paper, it was ensured that the combined storage capacity of all the UAVs was sufficient to accommodate the environment data at all locations of interest.

Further, in Manathara et al.’s paper, the positions of the fixed position targets were also not known in the polynomial time coalition formation algorithm (PTCFA) and optimal coalition formation algorithm (OCFA). If the targets are dynamic and moving all the time, one could understand the need for searching for them. However, if the targets are fixed and static, then the actual positions of the targets can be communicated to the UAVs at the start of the mission. This is a more realistic representation of problems that involve targets at fixed positions. The PTCFA and OCFA solutions proposed by Manathara et al., where targets are searched for, can easily be remodeled as a solution where the positions of the targets are known to the UAVs at the start of the execution of the algorithm by ensuring that the sensor range is large enough to cover the entire fly zone. The length of the diagonal of the square search area with each side 1000 m long is 1414 m. Fitting the UAVs with sensors that have a sensor range of 1500 m will ensure that the entire search area is covered and that all targets or the positions of environment data locations are detectable by the UAV sensors when the coalition formation algorithm is executed.

In [40], the mission completion time is minimized by selecting coalition paths of minimal length to each target using a coalition of minimal size. Manathara et al. described the Dubins longest path using three angles, $\theta_{1}$, $\theta_{2}$, $\theta_{3}$, and the radius, as indicated in Section 4. The θ’s are computed using the initial position of the UAV, the position of the target and the initial direction of flight of the UAV. Finding accurate values for $\theta_{3}$ may pose a problem as indicated in Section 4, and a different and more robust approach is adopted in this paper to calculate the Dubins longest path based on the following four attributes, (1) the initial position of the UAV, (2) the size of the angle traversed during the circular path, (3) the exit point where the UAV leaves the circular path and (4) the incoming angle of the UAV when approaching the target.

Coalition formation is considered using three approaches as described below.

The Dubins longest path was included in three algorithms developed by Manathara et al. as described in [40]. The algorithms are:

1. polynomial time coalition formation algorithm (PTCFA),
2. optimal coalition formation algorithm (OCFA) and
3. particle swarm optimization (PSO).

In all three algorithms, it is assumed that all UAVs fly at a constant speed and at a constant altitude. A short description of each algorithm appears below.

### 3.1. PTCFA

The PTCFA consists of two parts. First, each UAV is in search of a target and is equipped with a sensor with limited range capability. As soon as a target is detected, its position and resource requirements are communicated to all UAVs. The UAV that detects the target is referred to as the coalition leader. All UAVs that are not in a coalition and that contain any of the resources required by the target calculate their estimated time of arrival (ETA) or cost and communicate this information together with the amount of resources on board the UAV to the coalition leader. The coalition leader sorts the ETAs received from all responding UAVs in ascending order and then adds the UAV closest to the target to the potential coalition and accumulates its resources. If the accumulated resources do not meet the target’s resource requirements, then the next closest UAV is also included in the coalition and its resources accumulated, as well. This process of adding a UAV with the next smallest ETA to the potential coalition continues until the resources accrued satisfy the resource requirements of the target. In the second part of the PTCFA, the coalition leader sorts the ETAs of the UAVs in the potential coalition in ascending order. Again starting with the UAV closest to the target, the coalition leader removes the resources of this UAV from the resources accumulated. If the remaining accumulated resources after removal still satisfy the target’s resource requirements, then the UAV under consideration is removed from the potential coalition; otherwise, its resources are added back to the accumulated resources. The UAV removed is redundant since its resources are not required for prosecution. The removal from the coalition of the next closest UAV is then considered. This process ends after all UAVs in the coalition have been considered for removal. The coalition leader then informs all remaining coalition members of (a) their inclusion in the coalition and (b) the ETA of the UAV furthest from the target. Coalition members then calculate revised radii that increase their ETAs at the target to match the ETA of the UAV furthest from the target.

### 3.2. OCFA

The first part of the OCFA is the same as the first part of the PTCFA. In the second part, the size of the coalition is minimized using mixed integer linear programming such as MATLAB’s bintprog. The coalition leader then informs all coalition members of (a) their inclusion in the coalition and (b) the ETA at the target of the UAV furthest from the target. Coalition members then increase their radii appropriately so that their distances to the target match that of the UAV furthest from the target.

### 3.3. PSO

Particle swarm optimization (PSO) has been used in [40] to select an optimal set of UAVs for a single known target, given a set of UAVs and their resources. PSO is a nature-based stochastic optimization algorithm that emulates the swarm behaviors of bird flocks [42]. PSO searches for an optimum solution by merely drawing the position of each particle in the swarm toward its own historical best position and toward the position of the historical best particle in a defined neighborhood [43]. The position and velocity of each particle are updated over time. Each particle in the swarm is N×M-dimensional, where *M* denotes the number of targets and *N* the number of UAVs. Each particle is a potential solution to the optimization problem. The position vector of a particle is constructed as follows. For each UAV, there is an *M*-dimensional vector where each element stores the preferred target to be visited by the UAV, e.g., vector (5,3,2,1) indicates the preference of the UAV to visit targets in the order 5, 3, 2 and 1. The *N* vectors, each of dimension *M*, of the UAVs are concatenated to form an N×M position vector. The position vector of each particle contains the preferences of all *N* UAVs in visiting the various targets. Each value in the position vector of a particle indicates the target to be visited and potentially prosecuted by a specific UAV, e.g., if element (2,3) in the position vector has a value of four, then it indicates that UAV Number 2 prefers Target Number 4 to be visited during the third sequence. To check whether a particle can successfully prosecute a target, say Target 4, during Sequence 2, proceed by identifying all UAVs that containing a value of four as their second vector element. All the UAVs identified form a potential coalition to prosecute Target 4. Next, accumulate the resources of all the UAVs in the potential coalition, i.e., the UAVs that prefer Target 4. If the combined resources of all the UAVs in the potential coalition satisfy the resource requirements of Target 4, then a coalition is possible, and the coalition time is calculated as the time taken by the UAV with the largest ETA to reach the target. If all targets can be prosecuted by a particle, then the sum of the coalition distances of all coalitions will be returned as the cost of the prosecution by the particle. A fitness function evaluates the cost of a particle. The cost of a successful mission is simply the sum of the coalition time required to prosecute each target. If all targets are not prosecuted by a particle, then a large cost, say infinity, is assigned as the particle’s fitness value. For more details of the fitness function, refer to [40]. To kick start the process, the position vector of the swarm is initialized to random values in the range [0, *M*], where zero indicates a search and a non-zero integer of the prospective target to be prosecuted; targets being numbered from 1–*M*. The PSO process ensures that the position vector yielding the best fitness and thus lowest coalition time is returned as the minimum or optimal value.

In the experiments reported in this paper, the resources referred to in Manathara et al. were replaced with environment data and the targets with locations of interest from whence environment data must be collected.

The next section discusses the Dubins longest path.

## 4. Calculating the Dubins Longest Path

Dubins curves can be used to calculate the shortest path to a target for a fixed-wing UAV. A Dubins curve consists of all combinations of arcs of minimal turning radius and a straight line segment that joins the two arcs. Six possible configurations were considered, namely LSL, LSR, RSL, RSR, RLR and LRL, where L and R represent a left and right turn, respectively, and S a straight line segment. In many search missions, fixed-wing UAVs are required only to fly a circular path followed by a straight line path to reach a target. No second arc is required. Hence, the original Dubins curves that consist of three components can be reduced to a curve with two components, in which case only two possible cases need to be considered, i.e., LS and RS.

**Figure 3:**
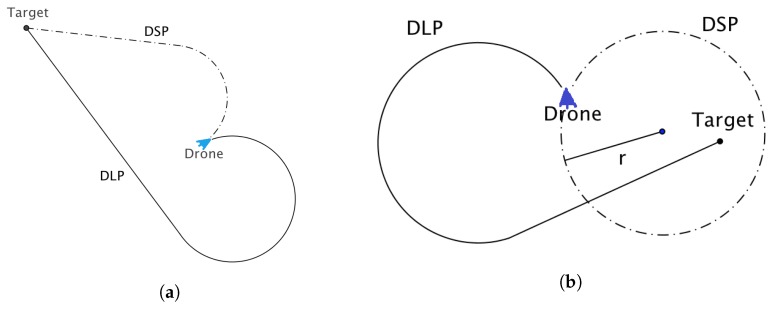
(**a**) Dubins shortest and longest paths (DSP and DLP, respectively) where the target lies to the left of the path of the drone; (**b**) DSP and DLP where the target lies to the right of the path of the drone.

Given a drone’s current position and a target, two types of Dubins paths can be traversed to reach the target, i.e., the Dubins shortest path (DSP) and Dubins longest path (DLP) (Figure 3a). A target may lie either to the left or to the right of the straight line path of a UAV. In the case of DSP, the UAV performs a turn in the same direction as the direction of the target relative to the UAV’s straight line path of flight. In the case of DLP, the UAV turns in the opposite direction of the direction of the target relative to a UAV’s position.

When the distance between the UAV and the target is smaller than the minimum turning radius of the UAV, then the Dubins shortest path cannot reach the target, as shown in Figure 3b. In this case, the UAV will simply circle around the target, never reaching it.

With the Dubins longest path, this problem does not exist, since the target is always reachable, irrespective of how close a target is to the UAV, as shown in Figure 3b.

To calculate the distance of the Dubins longest path, the following two cases are considered,

1. the target lies to the right of the straight line path of the UAV or
2. the target lies to the left of the straight line path of the UAV.

In Case (1), the UAV has to turn left or counterclockwise, while in Case (2) the UAV has to turn right or clockwise to reach the target.

**Figure 4:**
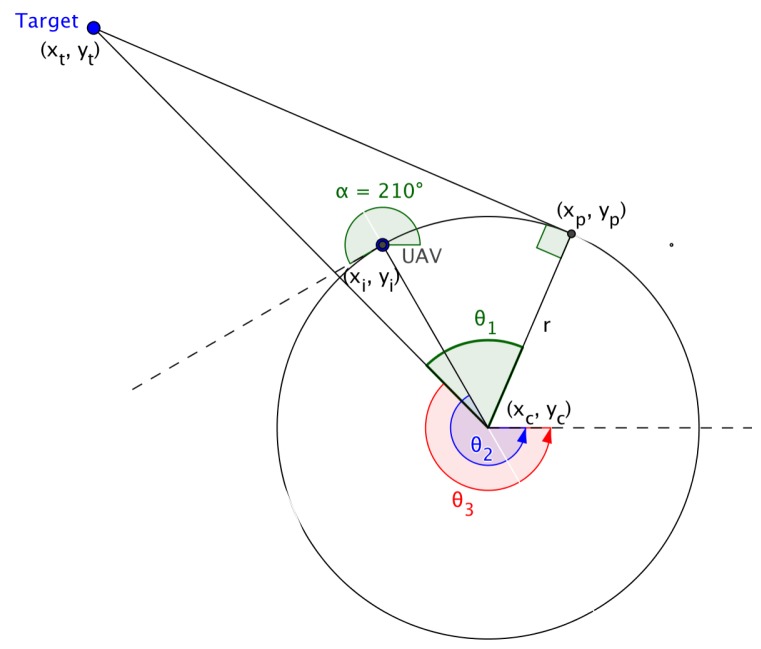
Dubins longest path.

In ‘Multiple UAV Coalitions for a Search and Prosecute Mission’ by Manathara et al., the estimated time of arrival of a UAV reaching its target based on the Dubins longest path for the two cases was defined in terms of three angles $\theta_{1}$, $\theta_{2}$ and $\theta_{3}$ (see Figure 4), where $\theta_{1}$ denotes the angle between the line that joins the center of the circle with the target and the line that joins the center of the circle with the exit point of the UAV from the arc, $\theta_{2}$ denotes the angle between the line that joins the initial position of the UAV with the center of the circle and the positive *x*-axis, measured in a positive direction, i.e., anti-clockwise, while $\theta_{3}$ denotes the angle between the line that joins the center of the circle with the target and the positive *x*-axis measured in a positive direction.

The angles are given by:

(17) $$\begin{array}{ccc} \theta_{1} & = & \arccos(\frac{\sqrt{{\left(x_{t}-x_{c}\right)}^{2}+{\left(y_{t}-y_{c}\right)}^{2}}}{r}), \end{array}$$

(18) $$\begin{array}{ccc} \theta_{2} & = & \frac{\pi }{2}-\alpha ,\;\;\mathrm{and} \end{array}$$

(19) $$\begin{array}{ccc} \theta_{3} & = & \arctan(\frac{y_{t}-y_{c}}{x_{t}-x_{c}}). \end{array}$$

In Case (1), the estimated time of arrival (ETA) was defined as,

(20) $$\begin{array}{ccc} D & = & \frac{r\left(2\pi -\left(\theta_{1}-\left(\theta_{2}-\theta_{3}\right)\right)\right)+r\tan\theta_{2}}{v_{i}}, \end{array}$$

and in Case (2),

(21) $$\begin{array}{ccc} D & = & \frac{r\left(2\pi -\left(\theta_{1}-\left(\pi -\theta_{3}\right)-\theta_{2}\right)\right)+r\tan\theta_{2}}{v_{i}}, \end{array}$$

where $v_{i}$ is the ground speed, $(x_{c},y_{c}$) is the center of the arc traversed by the UAV, $\left(x_{t},y_{t}\right)$ is the position of the target and α the angle of direction of flight of the UAV measured from the positive *x*-axis.

**Figure 5:**
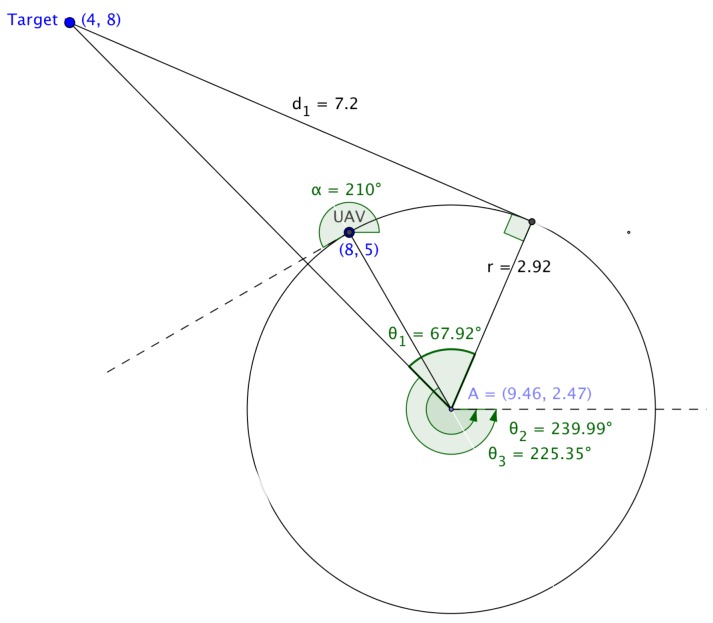
Calculating θ’s based on Manathara et al.

Calculating the Dubins longest path using Equations (10) and (11) may pose a problem in certain cases, as reflected in Figure 5.

Substituting the radius of the arc, the coordinates of the target’s position, the center of the arc and the angle of the flight of the UAV in Equations (7)–(18) yield values for $\theta_{1}$, $\theta_{2}$ and $\theta_{3}$ as $67.92^{{}^{\circ}}$, $-120.0^{{}^{\circ}}$ and $-45.65^{{}^{\circ}}$, respectively, while values computed using the mathematical tool Geogebra for $\theta_{1}$, $\theta_{2}$ and $\theta_{3}$ are $67.92^{{}^{\circ}}$, $239.99^{{}^{\circ}}$ and $225.35^{{}^{\circ}}$, respectively. This discrepancy resulted in an alternative approach to be explored to determine the Dubins longest path.

An alternative approach to calculate the Dubins longest path is proposed next. If the UAV’s initial position, $\left(x_{i},y_{i}\right)$, the angle of flight of the UAV, α, the target’s position $\left(x_{t},y_{t}\right)$ and the minimum turning radius of the UAV, $r_{min}$, are provided, then the distance of the circular path and the straight line path of Dubins longest path can be calculated based on the following data:

- center of the circular path formed when the UAV turns,
- the exit point of the UAV from the circular path,
- the central angle traversed by the UAV while turning and
- the angle of the direction of flight of the UAV when approaching the target along the straight line path.

The distance of the Dubins longest path is then simply computed as the distance traversed along the arc plus the distance covered by the straight line path that joins the exit point on the arc with the target, as shown in Equation (38). In the next section, each of the data values referred to above will be calculated.

### 4.1. Calculating the Coordinates of the Center of the Circle

The center of the circle is calculated for each of the two cases referred to above.

**Figure 6:**
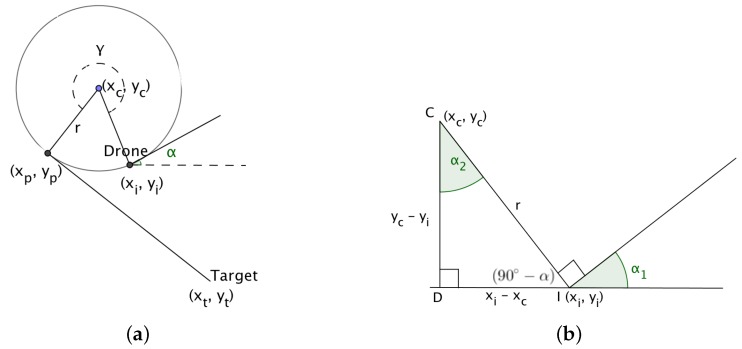
(**a**) Calculating the center of the circle when the target lies to the right of the path of the drone; (**b**) Calculating the center of the circle given the initial position, angle of the drone and the radius.

If the target lies to the right of the straight line path of the UAV, then the center of the circle, $\left(x_{c},y_{c}\right)$ is calculated as follows,

In triangle CDI , in Figure 6,

(22) $$\begin{array}{ccc} \frac{x_{i}-x_{c}}{r} & = & \sin\alpha_{2}\;\mathrm{and}\\ x_{c} & = & x_{i}-r\cdot \sin\alpha_{2}, \end{array}$$

and,

(23) $$\begin{array}{ccc} \frac{y_{c}-y_{i}}{r} & = & \cos\alpha_{2}\;\mathrm{and}\\ y_{c} & = & y_{i}+r\cdot \cos\alpha_{2}. \end{array}$$

When the target lies to the left of the straight line path of the UAV, then it can be shown that,

(24) $$\begin{array}{ccc} x_{c} & = & x_{i}+r\cdot \sin\alpha \;\mathrm{and} \end{array}$$

(25) $$\begin{array}{ccc} y_{c} & = & y_{i}-r\cdot \cos\alpha , \end{array}$$

where *r* is the radius of the arc and α denotes the angle of the direction of the UAVs initial flight path.

### 4.2. Calculating the UAV’s Exit Point on the Arc

Let $m_{k}$ denote the gradient of line $L_{k}$ in Figure 10, then:

(26) $$\begin{array}{cc} m_{2} & =\tan\;\alpha_{2}. \end{array}$$

If line $L_{2}$ is not parallel to the *y*-axis, then its equation is,

(27) $$\begin{array}{ccc} y & = & m_{2}\cdot x+c_{2}. \end{array}$$

Since $\left(x_{t},y_{t}\right)$ lies on line $L_{2}$, $\left(x_{t},y_{t}\right)$ can be substituted in Equation (27) to calculate the constant $c_{2}$, i.e.,

(28) $$\begin{array}{ccc} c_{2} & = & y_{t}-m_{2}\cdot x_{t}. \end{array}$$

Equation (27) can now be written as,

(29) $$\begin{array}{ccc} y & = & m_{2}\cdot x+\left(y_{t}-m_{2}\cdot x_{t}\right). \end{array}$$

Since line $L_{3}$ is perpendicular to line $L_{2}$, this implies:

(30) $$\begin{array}{ccc} m_{3}\cdot m_{2} & = & -1,\\ m_{3} & = & \frac{-1}{m_{2}}=cot\;\alpha_{2},\\ & = & \frac{y-y_{c}}{x-x_{c}},\\ y-y_{c} & = & m_{3}\cdot \left(x-x_{c}\right). \end{array}$$

The equation of $L_{3}$ is,

(31) $$\begin{array}{ccc} y & = & y_{c}+m_{3}\cdot \left(x-x_{c}\right). \end{array}$$

The point of tangency is the intersection of lines $L_{2}$ and $L_{3}$ in Figure 10 and is calculated as,

(32) $$\begin{array}{ccc} \left(x_{p},y_{p}\right) & = & (\frac{y_{c}-m_{3}\cdot x_{c}-c_{2}}{\;m_{2}-m_{3}},m_{2}\cdot x_{p}+c_{2}). \end{array}$$

If line $L_{2}$ is parallel to the *y*-axis, then line $L_{3}$ (that is perpendicular to $L_{2}$) is parallel to the *x*-axis; their corresponding equations are,

$$\begin{array}{cc} L_{2}: & x=x_{t},\\ L_{3}: & y=y_{c} \end{array}$$

and the point of tangency is,

(33) $$\begin{array}{ccc} \left(x_{p},y_{p}\right) & = & (x_{t},y_{c}). \end{array}$$

Similarly, if line $L_{2}$ is parallel to the *x*-axis, then line $L_{3}$ is parallel to the *y*-axis; the corresponding equations are,

$$\begin{array}{cc} L_{2}: & y=y_{t},\\ L_{3}: & x=x_{c} \end{array}$$

and the point of tangency is,

(34) $$\begin{array}{ccc} \left(x_{p},y_{p}\right) & = & (x_{c},y_{t}). \end{array}$$

The exit point of the UAV from the circular path is given by $\left(x_{p},y_{p}\right)$.

### 4.3. Calculating the Angle Traversed by the UAV While Turning

**Figure 7:**
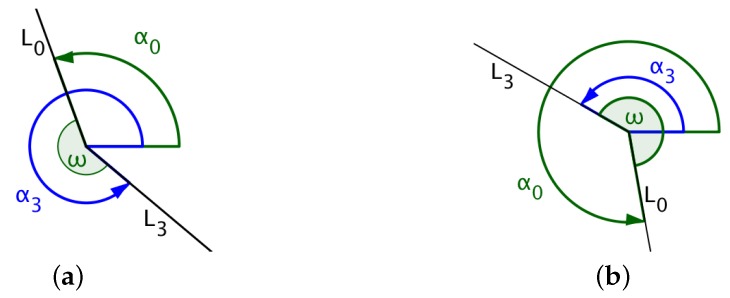
(**a**) UAV flies counterclockwise where $\alpha_{0}<\alpha_{3}$; (**b**) UAV flies counterclockwise where $\alpha_{0}>\alpha_{3}$.

**Figure 8:**
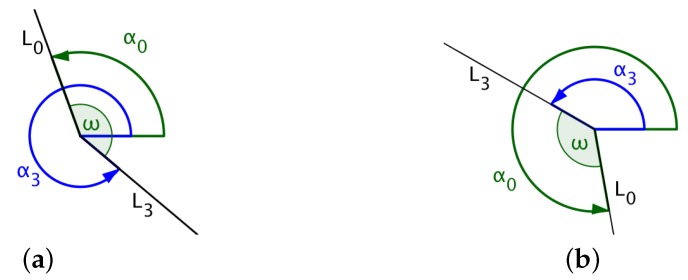
(**a**) UAV flies clockwise where $\alpha_{0}<\alpha_{3}$; (**b**) UAV flies clockwise where $\alpha_{0}>\alpha_{3}$.

If $L_{0}$ denotes the line that joins the UAV’s initial position $\left(x_{i},y_{i}\right)$ with the center of the circle $\left(x_{c},y_{c}\right)$ and $L_{3}$, the line that joins the exit position of the UAV on the arc $\left(x_{p},y_{p}\right)$ with the center of the circle, then the angle traversed by the UAV is the angle measured from line $L_{0}$ to line $L_{3}$ in the same direction as that of the UAV’s circular direction of flight. In Figure 7, $\alpha_{0}$ and $\alpha_{3}$ denote the angles formed between the *x*-axis and lines $L_{0}$ and $L_{3}$, respectively. The size of the angle traversed by the UAV for the circular path is the angle ω measured from $L_{0}$ to $L_{3}$, as shown in the diagrams in Figure 7 and Figure 8.

The central angle is calculated as indicated in Algorithm 1.

**Algorithm 1:** Calculating the central angle, ω.
 **if** (UAV turns left) **then**  **if** ($\alpha_{3}>\alpha_{0}$) **then**   $\omega \leftarrow \alpha_{3}-\alpha_{0}$  **else**   $\omega \leftarrow 360^{{}^{\circ}}+\left(\alpha_{3}-\alpha_{0}\right)$  **end if** **else**  **if** (UAV turns right) **then**   **if** ($\alpha_{0}>\alpha_{3}$) **then**    $\omega \leftarrow \alpha_{0}-\alpha_{3}$   **else**    $\omega \leftarrow 360^{{}^{\circ}}+\left(\alpha_{0}-\alpha_{3}\right)$   **end if**  **else**   $\omega \leftarrow 0^{{}^{\circ}}$  **end if** **end if**


### 4.4. Calculating the Incoming Angle of the UAV

**Figure 9:**
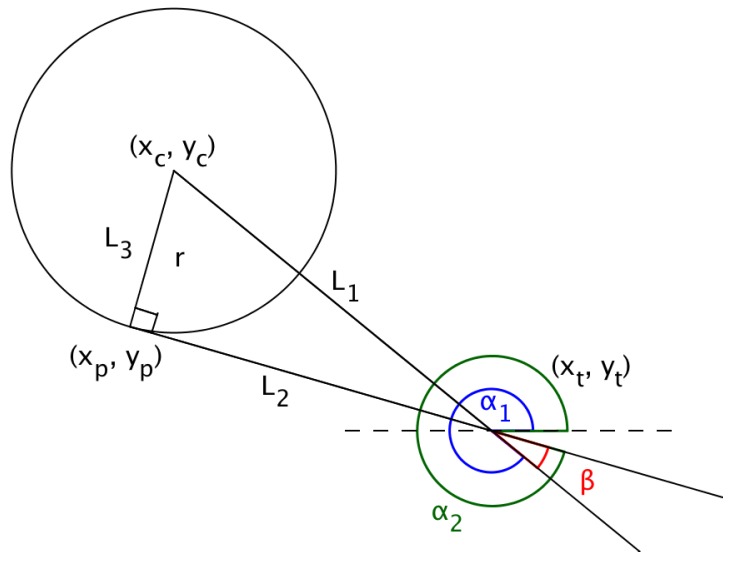
Target lies to the right of the UAV’s straight line path.

In the case where the target lies to the right of the straight line path of the UAV and the UAV has to turn anti-clockwise as reflected in Figure 9, let the new angle of the direction of the flight of the UAV be denoted by $\alpha_{2}$. If $\alpha_{1}$ is the angle between the extended hypotenuse and the positive *x*-axis and β is the angle between the hypotenuse extended and the tangent to the circle at $\left(x_{p},y_{p}\right)$ extended, then $\alpha_{1}$ and β can be calculated as follows,

(35) $$\begin{array}{ccc} \alpha_{1} & = & \arctan2(y_{t}-y_{c};x_{t}-x_{c}), \end{array}$$

(36) $$\begin{array}{ccc} \beta & = & \arcsin(\frac{r}{\sqrt{{\left(x_{t}-x_{c}\right)}^{2}+({\left(y_{t}-x_{c}\right)}^{2}}}), \end{array}$$

where arctan2(·,·) returns the angle measured between the positive *x*-axis and the line that joins the two points $\left(x_{t},y_{t}\right)$ and $\left(x_{c},y_{c}\right)$. If the resulting value is negative, then simply add $360^{{}^{\circ}}$ to yield the angle measured counterclockwise from the positive *x*-axis.

**Figure 10:**
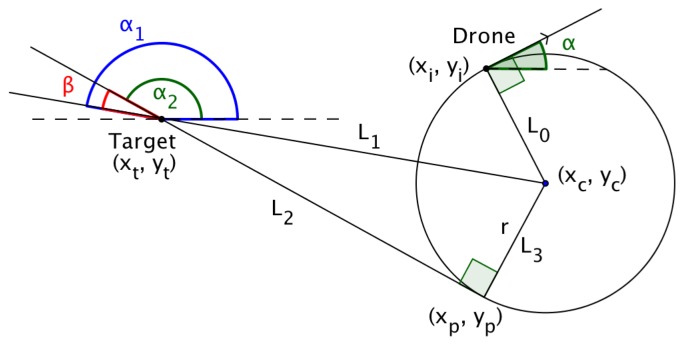
Target lies to the left of the UAV’s straight line path.

In Figure 9 and Figure 10, $L_{1}$ denotes the line that joins the center of the circle with the target, while $L_{2}$ denotes the line that joins the UAVs exit point, after turning, with the target. $L_{3}$ denotes the line that joins the center of the circle with the exit point.

In Figure 9, where the target lies to the right of the straight line path of the UAV resulting in the UAV turning left, the new incoming angle of the UAV is calculated as,

(37) $$\begin{array}{ccc} \alpha_{2} & = & \alpha_{1}+\beta . \end{array}$$

In the case where the target lies to the left of the straight line path of the UAV, as shown in Figure 10, the new angle of the UAV, $\alpha_{2}$, is calculated as,

(38) $$\begin{array}{ccc} \alpha_{2} & = & \alpha_{1}-\beta . \end{array}$$

Finally, the distance of the Dubins longest path, *d*, is the distance measured along the arc from the UAV’s initial position to the exit point on the circular path, plus the distance of the straight line path from the exit point to the target’s position, i.e.,

(39) $$\begin{array}{ccc} d & = & \omega \cdot r_{min}+\sqrt{{\left(x_{t}-x_{p}\right)}^{2}+{\left(y_{t}-y_{p}\right)}^{2}}. \end{array}$$

This concludes the discussion on the calculation of the Dubins longest path based on elementary geometry.

## 5. Experiments and Results

For each of the OCFA, PTCFA and PSO algorithms, 100 simulations were performed on a square search area with sides of length 1000 m. To restrict the UAVs to the search area and to allow sufficient space for turning around within the search area, a strip of width 100 m around the boundary of the search area was deemed out of place for placing the data collection locations. Locations were randomly placed at positions within the 800m×800m area inside the search area. The UAVs were restricted to flying inside this smaller area. The UAVs were also initially placed at random positions inside the fly zone with an angle of direction of flight generated randomly. All UAVs had a minimum turning radius of 50 m. A UAV that flies outside the fly zone will immediately turn around and can comfortably return inside the 100-m strip without leaving the search space. All UAVs were flying at a constant speed of 10 m/s. It is assumed that all UAVs fly at the same constant speed at the same altitude during the entire mission and that each UAV can store three different types of environment data.

The quantity for each type of environment data at each location of interest was generated as a random integer value in the range 0–3. The algorithmic parameters were set to the same values as in Manathara et al.’s paper, except that the storage capacity for each type of environment data of the UAVs was randomly generated with values ranging from 0 to the (number-of-locations-of-interest/2) and stored only if, for each type of environment data, the total sum of storage capacity for that specific type of data of all UAVs was equal to or greater than the total sum of quantities of the specific environment data of all the locations. This was done to ensure that the combined storage capacity of all the UAVs was sufficient to store all the environment data to be collected at the various locations.

In the simulations reported in this paper, the mission completion time, and not the coalition path length, was used as the cost. Simulations were performed for each of 5, 10, 15 and 20 locations of interest or targets with varying numbers of UAVs ranging from 5–20. The following values were recorded: mission completion time, i.e., time taken from the start of a mission until all the environment data of the last location were collected, total coalition time, i.e., the sum of the coalition time of all locations, and simulation time, i.e., the time taken to complete a simulation from the time when the UAVs were set in motion until the time when all the environment data of the last location of interest were collected.

### 5.1. Comparing Simulation Times of Algorithms Whose Cost Is Based on Either Coalition Time or on Mission Time

In [40], Manathara et al. used coalition time as the cost for minimization in their algorithms. Results reported in this section are based on the algorithms using mission time instead, as the cost for minimization. However, since coalition path length was key for the inclusion of a UAV in a potential coalition in both PTCFA and OCFA, no differences were observed in the results when either coalition time or mission time was considered as the cost in Table 1. However, a difference was observed between the simulation times of PTCFA and OCFA for each of the two types of cost approaches. Of the 16 experiments conducted in the case of OCFA, only two cases with mission time as the cost produced smaller simulation times. PTCFA with mission time as the cost achieved smaller simulation times than PTCFA with coalition time as the cost in 11 of the 16 cases. PSO with mission time as the cost reflected smaller simulation times than PSO with coalition time as the cost in all cases, bearing in mind that PSO takes the longest to execute of all three algorithms.

### 5.2. Effect of Increased Sensor Range

**Figure 11:**
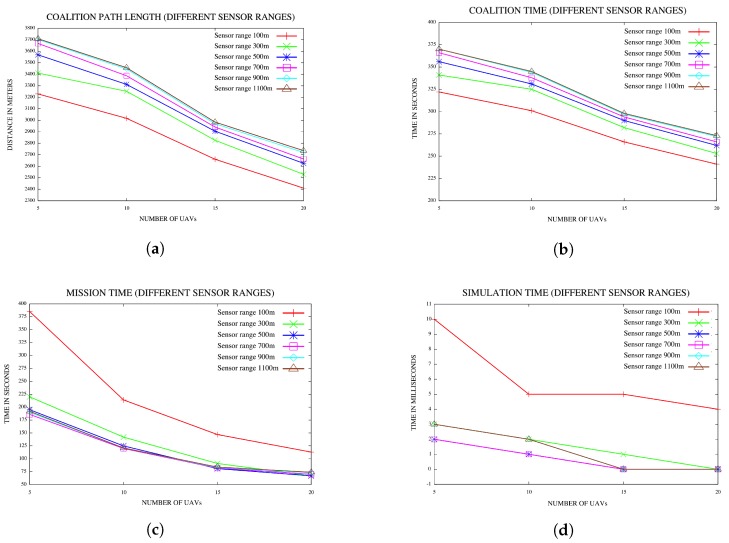
(**a**) Coalition path lengths for various sensor ranges; (**b**) coalition times for various sensor ranges; (**c**) mission time for various sensor ranges; (**d**) simulation time for various sensor ranges.

In [40], Manathara et al. compared the performances of PTCFA and OCFA with PSO. In their paper, the positions of the targets were not known to the UAVs for both PTCFA and OCFA, while the positions of the targets were known to the UAVs in PSO. In such a comparison, PSO has an unfair advantage over both PTCFA and OCFA, since the latter two algorithms must first search and find a target before they can form a coalition. Searching for a target consumes additional time, while PSO does not spend any time in searching for the targets and can immediately proceed with coalition formation, if the target’s resource requirements are met. A large sensor range that covers the entire search area enables a UAV to detect the positions of all the targets or locations of interest. In the second set of experiments, the sensor range of the UAVs was gradually increased from 100 m–1100 m in increments of 200 m; noting that locations are placed inside a square with each side 800 m long. A sensor range of 1100 m effectively covers a total distance of 2 × radius (= 2200 m), which is the diameter of the circle of the area covered by a sensor with a sensor range of 1100 m. A distance of 1100 m should cover almost the full distance of 1131 m between any two corners positioned diagonally opposite each other in the fly zone. A sensor range of 1100 m should enable the sensor of a UAV to detect all the locations of interest. A hundred simulations were performed with each sensor range value. Results are reported in Figure 11a–d.

Plots in Figure 11a indicate that coalition path length increases with an increase in sensor range. It should be noted that coalition path length refers only to the path length of the largest Dubins longest path to reach a target. The coalition path length does not include the distance covered by the UAV while searching for a target. With a small sensor range, coalition formation can only be initiated when a target is detected within this smaller range; hence, coalition formation is only triggered when the distance to a target is within the distance covered by the smaller sensor range. In the case of a larger sensor range, coalition formation is initiated similarly only when the distance between a UAV and a target falls within the larger sensor range. With a larger sensor range, coalition formation can be initiated much sooner than the case with a smaller sensor range. In the case of the larger sensor range, the coalition leader that detects the target might be the closest to the target with all other UAVs that join the coalition to be further from the target. Hence, generally, a larger sensor range will lead to larger coalition path lengths.

Since all UAVs fly at a constant speed and coalition time is calculated as coalition path length divided by the speed of the UAV, one would expect the coalition time to also increase with an increase in sensor range, as reflected in Figure 11b.

Mission time consists of time spent on searching for the locations of interest or targets and time spent on executing a coalition. During the execution of a coalition, the UAVs traverse the Dubins longest paths to reach the target, with the radii of the participating UAVs increased to ensure that all UAVs reach the target at the same time. With a smaller sensor range, targets are only detected once they are within this smaller sensor range of a UAV. Often, UAVs could find themselves flying away from a target that is just outside the sensor range and move effectively further away form a target while searching for a target. This is time wasted by the UAV that could have better been utilized by executing a coalition. With a larger sensor range, less time is spent and wasted on searching for targets, and coalitions are formed earlier, which leads to reduced mission times. The plots in Figure 11c confirm that mission time decreases with an increase in sensor range and that mission time decreases with an increase in the number of UAVs.

Simulation time decreases with an increase in sensor range, as reflected in Figure 11d. A sensor range of 500 m covers a circle with a corresponding diameter of 1000 m, which covers a large part of the square fly zone with each side length being 800 m. The plots in Figure 11d indicate that sensor ranges 500 m and 700 m produce identical simulation times, which on their face, implies that PTCFA did not benefit at all from the larger sensor range. However, Figure 11c puts this misleading conclusion to rest, since a sensor range of 700 m produced smaller mission times than a sensor range of 500 m. One can conclude that the benefits and losses with respect to simulation time in the case of 500 m and 700 m sensor ranges offset one another, resulting in similar simulation times. Similar simulation times are reported also for sensor ranges of 500 m, 700 m and 900 m in the case of 15 and 20 UAVs.

### 5.3. Effect of Increased Number of Types of Resources or Environment Data

**Figure 12:**
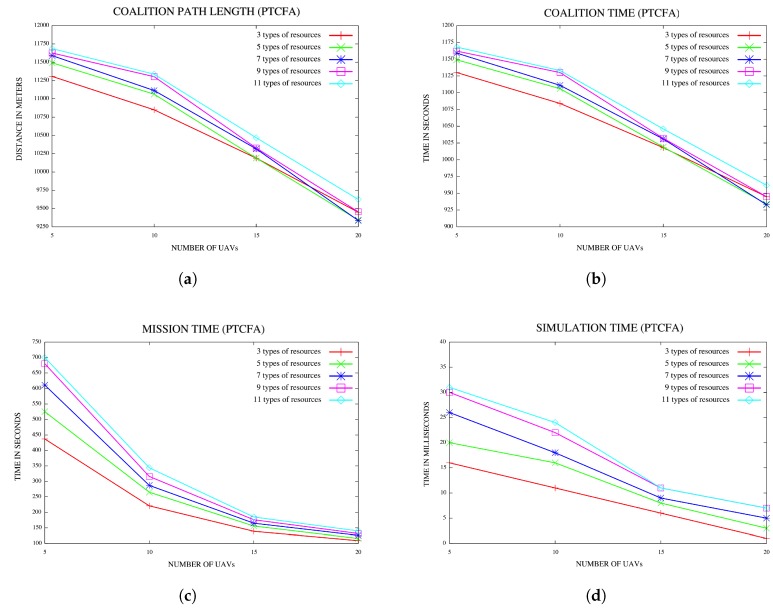
(**a**) Coalition path lengths for various numbers of resources for 15 locations; (**b**) coalition times for various numbers of types of resources for 15 locations; (**c**) mission time for various numbers of types of resources for 15 locations; (**d**) simulation time for various numbers of types of resources for 15 locations.

Another set of experiments was conducted to investigate the effect of an increased number of types of resources or environment data on the mission, w.r.t. coalition time, mission completion time and simulation time. The number of types of resources was increased from 3–11 in increments of two. Configurations comprised of 15 locations of interest or targets and UAVs varying from 5–20 in increments of five were considered. For each configuration, a hundred simulations were performed, and the average for each of the characteristics referred to above were recorded. Results are reported in Figure 12a–d. The plots in Figure 12a indicate that the coalition path length increases with an increase in the number of types of resources or environment data. A similar trend is noticed with the coalition time as reflected in Figure 12b. For each configuration of the number of environment data or resources, the coalition time decreases with an increase in the number of UAVs in both Figure 12a,b.

The plots in Figure 12c indicate that the mission time increases with an increase in the number of types of resources or environment data. For a fixed number of types of resources or environment data, the mission time decreases with an increase in the number of UAVs.

Graphs in Figure 12d indicate that simulation time also increases with an increase in the number of types of resources or environment data.

### 5.4. Comparing PSO with PTCFA and OCFA

**Figure 13:**
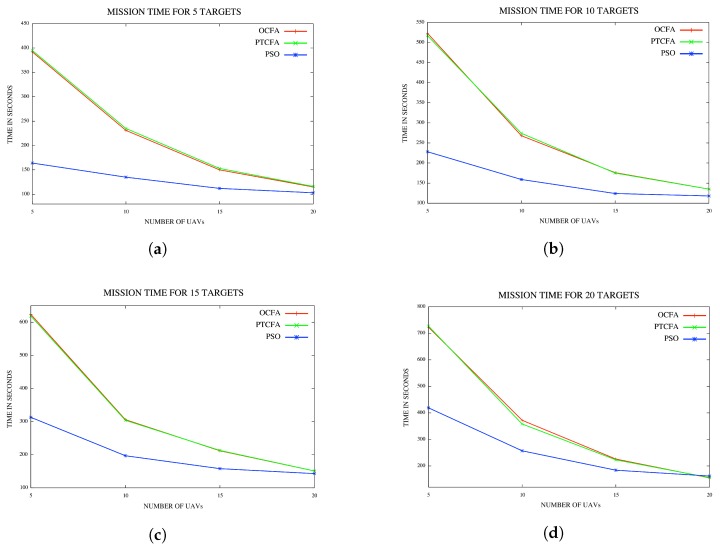
(**a**) Mission time for five targets/locations; (**b**) mission time for 10 targets/locations; (**c**) mission time for 15 targets/locations; (**d**) mission time for 20 targets/locations.

**Figure 14:**
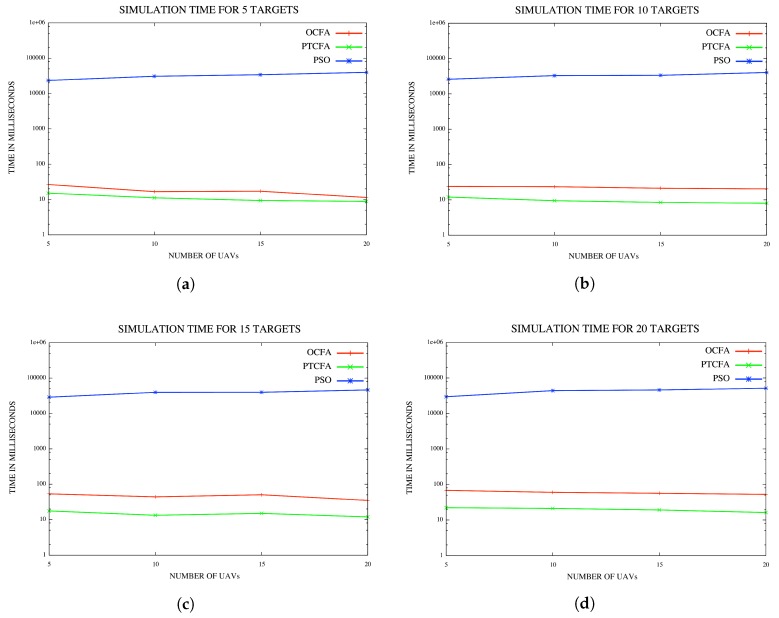
(**a**) Simulation time for five targets/locations; (**b**) simulation time for 10 targets/locations; (**c**) simulation time for 15 targets/locations; (**d**) simulation for time 20 targets/locations.

In the last set of experiments, the performance of PSO was compared to that of PTCFA and OCFA. For a fair comparison, sensor range was set to 1500 m to ensure that all targets or locations of interest were detectable from any position in the fly zone with the execution of PTCFA and OCFA. Average mission times and simulation times are reported for various configurations of UAVs and targets or locations of interest in Figure 13a and Figure 14d.

#### 5.4.1. Average Mission Time

The mission times of PSO, PTCFA and OCFA are reflected in Figure 13a–d. Results indicate that PSO had the best mission time of all three algorithms. PTCFA and OCFA had similar performances, with PTCFA achieving slightly better mission times than OCFA at times in Figure 13a–c. A possible explanation is that OCFA is optimized per target/location, and occasionally, the inclusion of the closest UAV in a coalition for a specific target/location may result in the distance to the next target/location being much further than had the UAV closest to the target/location been excluded from the first coalition. The OCFA algorithm optimizes a coalition w.r.t. a single target/location, and the solution proposed may not necessarily be an optimal solution across all targets.

Figure 13d reflects coalition path lengths, coalition times, mission times, coalitions and simulation times for a single run using five targets/locations and five UAVs for PTCFA and OCFA. Note, coalitions comprising different coalition members were formed for PTCFA and OCFA. PTCFA produced smaller coalition path lengths and mission completion times than OCFA. The simulation time of PTCFA was also much smaller than that of OCFA. 

Results for a single run are recorded in Table 2. Note the various coalitions formed for PTCFA and OCFA. Results show that different coalitions were formed by the two algorithms. Although OCFA had a lower mission time than PTCFA, its coalition path lengths and simulation times were higher than those of PTCFA. PTCFA produced smaller mission times than OCFA in 10 out the 100 simulations.

#### 5.4.2. Average Simulation Time

The plots in Figure 14a–d indicate that PSO took the longest simulation time of all three algorithms. In the case of PSO, the average simulation time also increased with an increase in the number of UAVs. PTCFA had the lowest simulation time of all the algorithms. The simulation time of OCFA was slightly longer than that of PTFCA, but much smaller than that of PSO. Results indicate that PTCFA with the shortest simulation time of all three algorithms is the best candidate for coalition formation since the computational overhead must be minimal.

#### 5.4.3. Energy Savings

In the experiments conducted by Manathara et al. in [40], data files were randomly populated and not initialized to ensure that the total sum of the quantities of resources of the UAVs was at least equal to the total sum of the quantities of the corresponding resource of all the targets. Their argument is that often, at the start of a search and prosecute mission, one is not aware of the resource requirements of the various targets. However, there are cases when the resource requirements are known at the start of a mission, e.g., the size of the data to be collected at various targets when performing a city’s remote sensing is known in advance. In these cases, one can ensure that the resource requirements are met before the mission is initiated. Furthermore, if the environment data of all the locations of interest cannot be stored in the UAVs, then the mission will not be completed successfully. These unsuccessful missions imply that energy was wasted while performing the various coalitions. To quantify the benefit in energy savings, 1600 data files were populated randomly without ensuring that the UAVs had sufficient storage capacity to accommodate all the environment data to be collected at the various locations. Of the 1600 data files, in 457 cases, the UAVs could not store all the environment data collected. For the experiments reported in this paper, it was ensured that the UAVs had sufficient storage capacity to store all the data collected. In the case of data files that were randomly populated, 28.6% (= 4571/600) would not lead to successful prosecution of the targets. The approach adopted in the experiments resulted in an energy savings of 71.4%.

## 6. Conclusions

The Dubins longest path can be used to describe the path traversed by a fixed-wing unmanned aerial vehicle in pursuit of a target. This paper provides an approach that calculates the Dubins longest path using elementary geometry. Experiments were conducted where the Dubins longest path was applied to algorithms that included particle swarm optimization, the polynomial time coalition formation algorithm and the optimal coalition formation algorithm. Simulation results indicated that although PSO had the lowest average mission completion time, and it had the longest simulation time of all three algorithms. The latter does not make PSO a good candidate for coalition formation for UAVs where minimal computational time is of paramount importance. PTCFA’s smaller simulation time than OCFA and its fairly reasonable mission time for a large number of UAVs compared to PSO make it a viable option as an algorithm for coalition formation for fixed-wing UAVs. Results confirm that for each type of algorithm, an increase in the number of UAVs led to a decrease in mission time. The constraint that UAVs have sufficient resources that meet the resource requirement of the targets led to a considerable savings in energy. An increase in the sensor range produced an increase in coalition time with a corresponding reduction in mission times. Results indicate that an increase in the number of types of resources led to an increase in coalition time and also to an increase in mission time. An increase in the number of UAVs that all contained the same number of types of resources assigned to a mission led to a decrease in mission time. Results also indicate that an increase in the number of UAVs led to an increase in simulation time in the case of PSO, but to a decrease in the case of PTCFA and OCFA.

For future work, the proposed model will be improved by reducing the assumptions. Environmental conditions such as the wind and weather need to be considered, as well as their impact on path planning and coalition formation. When many UAVs must be used in a relatively small area, collision avoidance will be of paramount importance for successful completion of a mission. Collision avoidance will be investigated and applied to the coalition formation algorithms used in this study. Experiments will be extended by considering heterogeneous UAVs, as well as the effect of different UAV specifications on the performance of coalition formation in data muling.

## Figures and Tables

**Table 1:** Simulation times in milliseconds of the optimal coalition formation algorithm (OCFA), the polynomial time coalition formation algorithm (PTCFA) and particle swarm optimization (PSO).

		OCFA		PTCFA		PSO	
No of Targets	No of UAVs	Based on Coalition Time	Based on Mission Time	Based on Coalition Time	Based on Mission Time	Based on Coalition Time	Based on Mission Time
5	5	19.17	19.95	12.62	8.61	22,414.8	21,570.1
5	10	12.98	15.67	8.30	4.89	34,006.9	32,734.6
5	15	9.67	10.76	7.39	5.14	43,106.9	405,858.5
5	20	8.87	9.53	7.21	19.25	52,920.2	49,756.5
10	5	16.36	17.46	13.34	19.00	24,591.3	23,793.2
10	10	16.55	16.33	12.45	16.81	40,502.0	37,648.8
10	15	14.43	15.06	11.38	10.37	49,174.7	47,941.2
10	20	13.97	15.34	11.41	9.54	59,669.6	57,586.5
15	5	24.89	28.07	24.60	20.63	28,950.8	24,245.7
15	10	19.66	21.77	21.66	16.95	59,202.1	45,361.7
15	15	22.98	25.71	19.68	21.32	71,315.7	55,908.2
15	20	22.32	29.67	16.88	22.53	88,065.8	66,963.6
20	5	30.23	33.67	27.69	27.07	31,280.2	20,218.5
20	10	27.10	31.03	26.23	26.19	50,740.8	50,217.7
20	15	25.47	28.18	24.31	22.56	62,134.9	60,292.0
20	20	24.43	24.29	19.63	18.81	72,133.8	71,825.6

**Table 2:** Comparison of a single execution between PTCFA and OCFA for 5 targets/locations and 5 UAVs.

**PTCFA**							
**Location or Target No.**	**Agents**	**Detected at Time in Seconds**	**Coalition Path Length in Meters**	**Coalition Time in Seconds**	**Coalition Leader**	**Mission Completion Time in Seconds**	**Simulation Time in Milliseconds**
4	0	0.0	362.44	36.2	0		
3	0, 2	60.7	564.76	56.5	2		
1	0	97.5	851.11	85.1	0	269.0	12.0
2	0, 3	195.0	446.48	44.6	3		
0	2, 1	212.7	841.71	84.2	1		
		Total	3066.5	306.65			
**OCFA**							
**Location or Target No.**	**Agents**	**Detected at Time in Seconds**	**Coalition Path Length in Meters**	**Coalition Time in Seconds**	**Coalition Leader**	**Mission Completion Time in Seconds**	**Simulation Time in Milliseconds**
4	0	0.0	362.44	36.2	0		
3	0, 2	60.7	564.76	56.5	2		
1	1	97.5	791.49	79.1	1	303.0	20.0
2	3, 4	189.1	657.18	65.7	4		
0	2, 1	212.7	903.14	90.3	1		
		Total	3279.01	327.9			
